# International Clinics

**Published:** 1901-10

**Authors:** 


International Clinics.—A Quarterly of Clinical Lectures and especially prepared articles of Medicine, Neurology, Surgery, Therapeutics, Obstetrics, Pediatrics, Pathology, Dermatology, Diseases of the Eye, Ear, Nose and Throat, and other topics of interest to students and practitioners. By leading members of the medical profession throughout the world. Edited by Henry W. Cottell, A. M., M. D., Philadelphia, U. S. A.; with the collaboration of John B. Murphy, M. D.; A. D. Blackader, M. D.; H. C. Wood, M. D.; T. M. Rotch, M. D.; E. Landolt, M. D.; Thos. G. Morton, M. D.; C. H. Reed, M. D. With regular correspondents in Montreal, London, Paris, Leipsic and Vienna. Vol. III. Tenth series, 1900. $2.00. Philadelphia: J. B. Lippincott Co. 1900.

This volume contains an article on "Aseptic Urethral Instrumentation," by Ferdinand C. Valentine, M. D., of New York, and is the kind of article that will benefit the general practitioner because it shows wherein failures follow hasty and unprepared operations, and why success comes from careful attention to asepsis.

There are twenty-seven other articles on various practical subjects, and are written by leading men all over the country. There are forty-five plates and figures used to illustrate the subject matter.

The International Clinics is deservedly a popular book.

